# Breast density quantification in dual‐energy mammography using virtual anthropomorphic phantoms

**DOI:** 10.1002/acm2.14360

**Published:** 2024-04-22

**Authors:** Gustavo Pacheco, Jorge Patricio Castillo‐Lopez, Yolanda Villaseñor‐Navarro, María‐Ester Brandan

**Affiliations:** ^1^ Instituto de Física Universidad Nacional Autónoma de México Mexico City Mexico; ^2^ Departamento de Imagen Instituto Nacional de Cancerología Mexico City Mexico

**Keywords:** clinical images, dual‐energy decomposition, dual‐energy mammography, magnetic resonance imaging, virtual phantoms, volumetric breast density

## Abstract

**Purpose:**

Breast density is a significant risk factor for breast cancer and can impact the sensitivity of screening mammography. Area‐based breast density measurements may not provide an accurate representation of the tissue distribution, therefore volumetric breast density (VBD) measurements are preferred. Dual‐energy mammography enables volumetric measurements without additional assumptions about breast shape. In this work we evaluated the performance of a dual‐energy decomposition technique for determining VBD by applying it to virtual anthropomorphic phantoms.

**Methods:**

The dual‐energy decomposition formalism was used to quantify VBD on simulated dual‐energy images of anthropomorphic virtual phantoms with known tissue distributions. We simulated 150 phantoms with volumes ranging from 50 to 709 mL and VBD ranging from 15% to 60%. Using these results, we validated a correction for the presence of skin and assessed the method's intrinsic bias and variability. As a proof of concept, the method was applied to 14 sets of clinical dual‐energy images, and the resulting breast densities were compared to magnetic resonance imaging (MRI) measurements.

**Results:**

Virtual phantom VBD measurements exhibited a strong correlation (Pearson's r>0.95) with nominal values. The proposed skin correction eliminated the variability due to breast size and reduced the bias in VBD to a constant value of −2%. Disagreement between clinical VBD measurements using MRI and dual‐energy mammography was under 10%, and the difference in the distributions was statistically non‐significant. VBD measurements in both modalities had a moderate correlation (Spearman's ρ= 0.68).

**Conclusions:**

Our results in virtual phantoms indicate that the material decomposition method can produce accurate VBD measurements if the presence of a third material (skin) is considered. The results from our proof of concept showed agreement between MRI and dual‐energy mammography VBD. Assessment of VBD using dual‐energy images could provide complementary information in dual‐energy mammography and tomosynthesis examinations.

## 1 INTRODUCTION

Breast density, defined as the proportion of fibroglandular tissue within the mammary gland, has been identified as a significant risk factor for breast cancer. Studies have consistently shown that women with higher breast density are at a greater risk of developing breast cancer than those with lower densities.^1^, ^2^ Visual assessment is the most widely used method for evaluating breast density, but it is subjective, susceptible to inter‐ and intra‐observer variability, and only provides an area‐based estimate of breast density. Furthermore, it has been found that cancer risk models perform better when implementing breast density as a continuous variable, as opposed to a visually‐assessed categorical scale.^3^ Consequently, there is a growing interest in measuring volumetric breast density (VBD), which considers the entire breast volume and provides a more accurate numerical assessment of the amount of fibroglandular tissue.

For VBD measurements, the breast is usually assumed to be composed of fibroglandular and adipose tissues. If the volumes of these two tissues are measured, then VBD is computed as the ratio of fibroglandular tissue volume to total volume. As mammography is the most prevalent screening modality for breast cancer, there is a special interest in VBD quantification techniques based on this modality, and Quantra^4^ and Volpara^5^ are commercially available tools able to evaluate VBD from single mammograms. These tools require modeling of the breast and estimation of parameters, such as total breast thickness, to infer the volumetric measurements, and uncertainties on these have been shown to impact VBD quantification.^6^

Alternatively, the combination of two mammography images acquired with different x‐ray spectra, known as dual‐energy mammography, enables the direct decomposition of the image into two of the materials that constitute the breast, and techniques based on the decomposition into fibroglandular and adipose tissue thicknesses have been proposed to obtain VBD,^7^, ^8^, ^9^, ^10^ however, their application in clinical images or anthropomorphic phantoms has been limited. Previous studies have mainly focused on assessing the accuracy of these methods using calibration or quality control phantoms,^11^, ^12^, ^13^ leaving a gap in our understanding of their performance in real‐world clinical settings. Furthermore, there is a lack of multimodality studies comparing VBD measured with dual‐energy to measurements obtained from 3D modalities such as magnetic resonance imaging (MRI) or computed tomography (CT), which provide a closer approximation to the volumetric distribution of breast tissue. To establish the viability of dual‐energy mammography for VBD quantification, it is important to ensure consistency with results from 3D modalities. Therefore, there is a need for further investigation and assessment of these methods in clinical images and anthropomorphic phantoms to evaluate their feasibility and accuracy for potential applications in breast imaging.

The goal of this work was to evaluate the dual‐energy decomposition method's performance in a set of heterogeneous virtual phantoms that reflect the clinical distribution of breast sizes and compositions. We assessed the method's intrinsic bias and variability using simulated anthropomorphic phantoms in which the true tissue distributions are known. The method was applied to clinical dual‐energy images acquired as part of a contrast‐enhanced mammography (CEM) protocol, a widely accepted technique to visualize angiogenesis in lesions.^14^ As a proof‐of‐concept, the dual‐energy mammography VBD measurements were compared with values obtained from segmented MRI volumes of the same breast.

## 2 MATERIALS AND METHODS

### 2.1 VBD quantification in dual‐energy breast images

The method was based on the dual‐energy material decomposition formalism,^15^, ^16^ and following common practice, we used fibroglandular tissue and adipose tissue as basis materials. Although a comprehensive description of the calibration procedure can be found in previous work,^17^ we provide a brief overview.

#### 2.1.1 Dual‐energy calibration

The dual‐energy calibration consisted of acquiring images of calibration phantoms with low and high x‐ray energies, and finding a mapping function that relates pixel values to material thickness in the phantoms.

As an initial step, images were converted to normalized signal intensity maps using a logarithmic transform. The normalization involved subtracting the air signal (average pixel value outside the phantom) from each pixel value in the images. Equation (1) shows the mapping function to calculate material thickness $t_{x}$ (x=g for fibroglandular tissue and x=a for adipose tissue), from the pixel values in the normalized low‐energy (LE) and high‐energy (HE) images ($S_{LE}$ and $S_{HE}$, respectively)

(1) $$\begin{array}{ccc} t_{x}\;\left(S_{LE},S_{HE}\right) & = & c_{1x}\;+c_{2x}S_{LE}+c_{3x}S_{HE}+c_{4x}S_{LE}S_{HE}\\ & & +c_{5x}{\left(S_{LE}\right)}^{2}+c_{6x}{\left(S_{HE}\right)}^{2}+c_{7x}{\left(S_{LE}\right)}^{3}\\ & & +c_{8x}{\left(S_{HE}\right)}^{3} \end{array}$$

The eight parameters in Equation (1) ($c_{1x}$ to $c_{8x}$) were fitted for each material (fibroglandular and adipose tissue). Though higher‐grade polynomials and rational functions have been used as mapping functions,^7^, ^16^ we opted for the lowest degree polynomial that produced an acceptable fitting accuracy. The criteria to accept the fit were $R^{2}$ values higher than 0.99 and mean residuals no larger than 5%.

**FIGURE 1:**
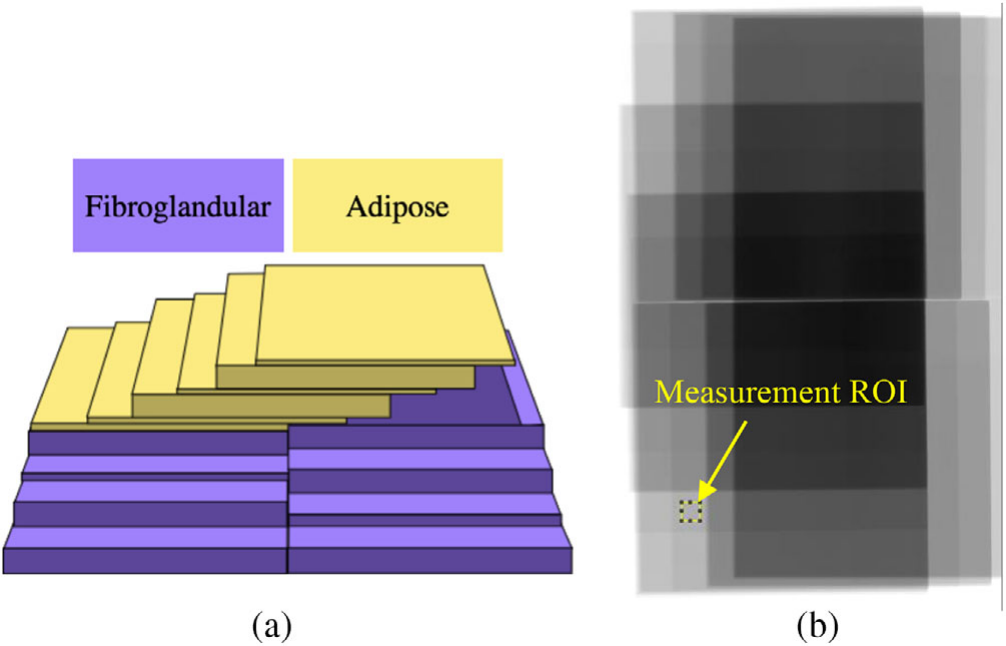
(a) Schematic representation of the calibration phantom. (b) Cropped low‐energy image of the calibration array and a selected 70 × 70 px region of interest (ROI), indicated by an arrow.

Our calibration phantoms consisted of orthogonal stair‐step arrangements of fibroglandular‐ and adipose‐tissue‐equivalent materials, as shown in Figure 1. Such configurations provide 43 unique pairs of material thicknesses within a range of values of clinical interest.

By applying Equation (1) to the dual‐energy image set, thickness maps $t_{g}\left(i,j\right)$ and $t_{a}\left(i,j\right)$ were obtained, where i and *j* are the pixel coordinates. The fibroglandular tissue volume $V_{g}$ was measured by integrating $t_{g}\left(i,j\right)$ over the breast area, and the total breast volume $V_{T}$, by integrating the total tissue thickness map $T\;\left(i,j\right)=t_{g}\;\left(i,j\right)+t_{a}\left(i,j\right)$. VBD was defined as $VBD\;=\;100\%(V_{g}/V_{T})$.

#### 2.1.2 Image processing

Our method for VBD quantification in breast images, either simulated or clinical, required two additional processing steps: the segmentation of the breast from the background and a correction for the skin covering the breast tissue. As all the images were craniocaudal projections, the segmentation was achieved by identifying the breast border using an adaptive thresholding approach. Although the segmentation successfully eliminated the skin at the border, the skin layers above and below the breast tissue still contributed to the tissue maps.

Equation (1) decomposes the contribution of the skin to the low‐energy and high‐energy signals into equivalent fibroglandular and adipose tissue thicknesses $t_{g}^{e}$ and $t_{a}^{e}$, respectively. These equivalent thicknesses do not correspond to actual fibroglandular and adipose tissue, constituting an additive bias in their quantification. We estimated $t_{g}^{e}$ and $t_{a}^{e}$ using the two‐material dual‐energy decomposition, which is equivalent to solving Equation (2) using different skin thicknesses $t_{sk}$.

(2) $$\left(\begin{array}{cc} \mu_{g}\left(E_{1}\right) & \mu_{a}\left(E_{1}\right)\\ \mu_{g}\left(E_{2}\right) & \mu_{a}\left(E_{2}\right) \end{array}\right)\;\left(\begin{array}{c} t_{g}^{e}\\ t_{a}^{e} \end{array}\right)=\left(\begin{array}{c} \mu_{sk}\left(E_{1}\right)t_{sk}\\ \mu_{sk}\left(E_{2}\right)t_{sk} \end{array}\right)\;$$

The linear attenuation coefficients of the fibroglandular tissue ($\mu_{g}$), adipose tissue ($\mu_{a}$) and skin ($\mu_{sk}$) were obtained using Hammerstein's elemental compositions.^18^ To account for the poly‐energetic nature of mammography spectra (see a representative pair of CEM spectra in fig. 2.6 of reference 14), Equation (2) was solved for every energy combination $\left(E_{1},E_{2}\right)$ in the relevant range for our acquisitions, with $E_{1}$ and $E_{2}$ ranging from 10 to 45 keV in 1 keV steps. The solutions were proportional to the skin thickness $t_{sk}$, that is $t_{g}^{e}\left(E_{1},E_{2}\right)\;=\;a\left(E_{1},E_{2}\right)t_{sk}$ and $t_{a}^{e}\left(E_{1},E_{2}\right)\;=\;b\left(E_{1},E_{2}\right)t_{sk}$ for $t_{sk}$ in the 0 to 5 mm range. The equivalent thicknesses were estimated by averaging $t_{g}^{e}\left(E_{1},E_{2}\right)$ and $t_{a}^{e}\left(E_{1},E_{2}\right)$ over the relevant energy combinations for our mammography spectra. For a given nominal skin thickness $t_{sk}$ the corresponding $t_{g}^{e}$ and $t_{a}^{e}$ were $1.36t_{sk}$ and $-0.44t_{sk}$, respectively. By subtracting $t_{g}^{e}$ and $t_{a}^{e}$, the skin contribution to the tissue maps was eliminated.

In both the phantom and clinical images, the skin above and below the breast tissue was approximated by a constant thickness layer. The simulated phantoms had an average skin thickness of 3.7 mm and the skin was approximated by a constant layer of that thickness. In the clinical images, a skin thickness of 3.5 mm was used. This value was based on measurements in segmented breast CT volumes^19^ and in vivo studies.^20^

### 2.2 Breast density quantification in virtual phantoms

We simulated 150 virtual anthropomorphic breast phantoms within the openVCT framework (University of Pennsylvania).^21^ These phantoms had randomized glandular tissue distributions and were divided into three breast volume categories: “large” (709 mL), “medium” (218 mL), and “small” (50 mL).

The simulated mammography system was based on the clinical unit (AMULET Innovality, FUJIFILM Corporation, Tokyo, Japan) commercialized for CEM. It features a tungsten anode, a 50 µm rhodium filter, and a 700 µm aluminum (Al) filter. To generate the dual‐energy images, we used the radiological technique previously optimized by Castillo et al. for CEM acquisitions.^22^ Low‐energy images were acquired at 31 kV using the rhodium filter, resulting in a spectrum with average energy of 19.2 keV and a half‐value layer (HVL) of 0.57 mm Al. High‐energy images were acquired at 45 kV with the aluminum filter plus an additional 5 mm of external aluminum filtering, resulting in an average energy of 33.3 keV and HVL of 2.55 mm Al. This choice of dual‐energy spectra is justified since, at present, CEM studies have gained wide acceptance and are being used extensively in clinical settings.

The output images of the virtual phantoms were logarithmically transformed to match the response of the Fujifilm system. The accuracy of the simulated images against the clinical system has been previously validated by Pacheco et al.^23^ by comparing the system modulation transfer function and pixel value response as a function of thickness and glandularity.

To generate the calibration dataset, the stair‐step phantom shown in Figure 1 was simulated using the same dual‐energy technique as the virtual phantoms. VBD was then measured in the images following the procedure described in Section 2.1, and the results were compared to the nominal values. The method's linearity, bias and variability were assessed following the Quantitative Imaging Biomarker Alliance technical recommendations.^24^

### 2.3 Proof of concept in clinical images and comparison to MRI measurements

As a proof of concept, we applied the dual‐energy decomposition method to patient images retrospectively collected from a previous research protocol.^22^ The protocol included 14 women with suspected multicentric breast cancer, and it involved the acquisition of various MRI sequences and a CEM study. In order to minimize the possible effect of the iodine presence in the mammography images, only the craniocaudal images of the breast contralateral to the lesion were analyzed. Both the patient and calibration dual‐energy image sets were acquired with the parameters described in Section 2.2. The clinical dual‐energy calibration involved acquiring images of the calibration phantom shown in Figure 1 and following the procedure detailed in Section 2.1. Both the patient and calibration dual‐energy image sets were acquired with the parameters described in Section 2.2.

The measured dual‐energy mammography‐based densities, $VBD_{DEDM}$, were compared to those obtained by processing MRI studies of the same breast, $VBD_{MRI}$. The processed MRI studies were acquired using the IDEAL (Iterative decomposition of water and fat with echo asymmetry and least‐squares estimation) sequence (GE Healthcare). As there is excellent contrast between fatty and non‐fatty tissue in the IDEAL MRI slices, adaptive thresholding approach^25^ was sufficient to adequately segment adipose from the rest, which is assumed to be fibroglandular. The goal of this comparison was to ensure that the VBD values derived from dual‐energy mammography fell within a clinically reasonable range.

Correlation between $VBD_{DEDM}$ and $VBD_{MRI}$ was assessed using Spearman's correlation coefficient (ρ). Agreement between both techniques was assessed using Wilcoxon's signed rank test on the difference distribution, as well as Bland‐Altman statistics. The p value significance for all tests was 0.05.

## 3 RESULTS

### 3.1 Breast density quantification in virtual phantoms

**FIGURE 2:**
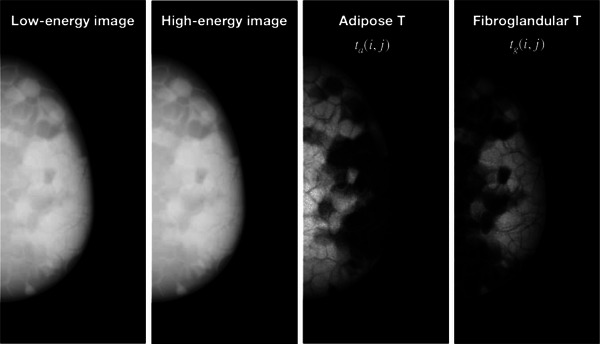
Dual‐energy decomposition of a simulated dual‐energy image pair. From left to right: LE image, HE image, adipose tissue map, and fibroglandular tissue map. The nominal and quantified VBD for this phantom were 42.9% and 40.5%, respectively.

Figure 2 shows a representative dual‐energy image pair of the virtual phantoms, and its decomposition into fibroglandular and adipose tissue maps (see Section 2.2).

**FIGURE 3:**
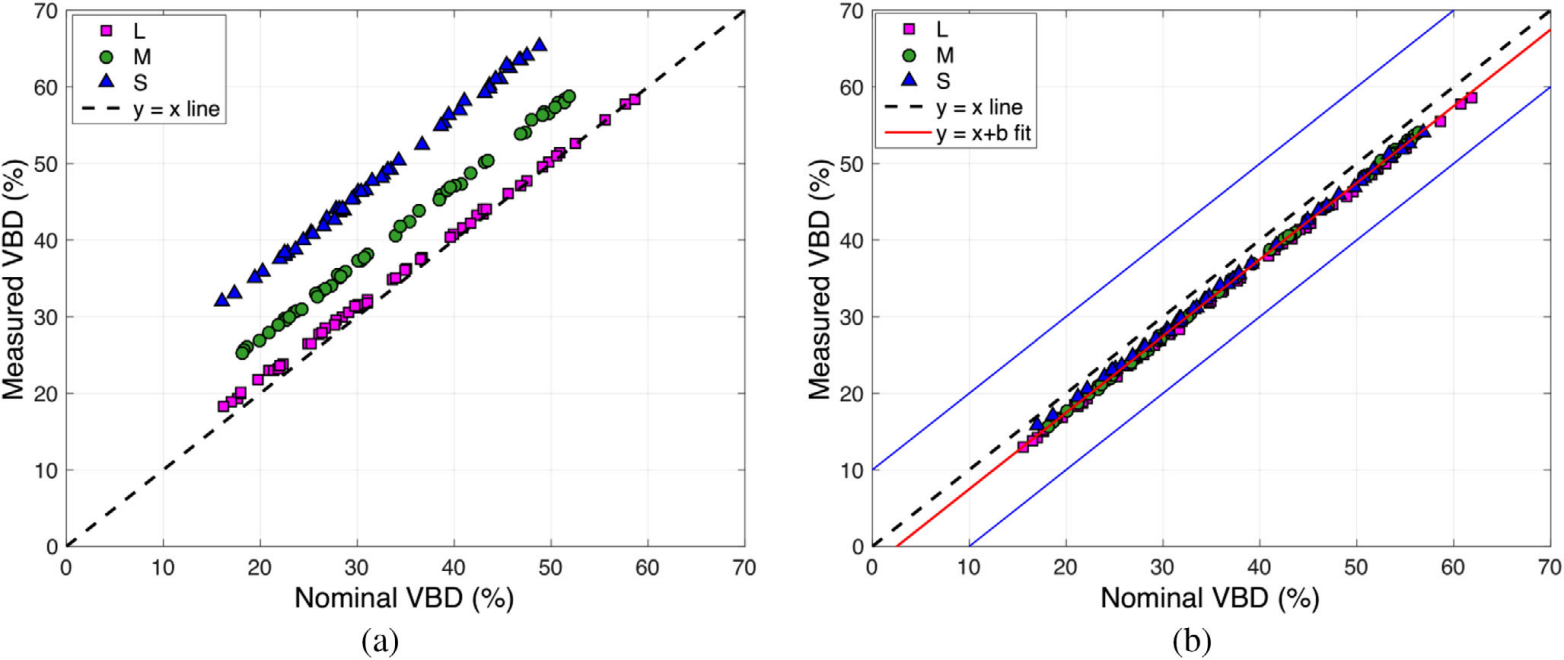
Simulated phantoms. (a) Measured VBD versus nominal VBD, no skin correction. The dashed black line is the identity. (b) Measured VBD versus nominal VBD, with skin correction. The dashed black line is the identity, the solid red line is the best fit of the data to a straight line, and the solid blue lines represent a ± 10% difference in VBD. Data are classified by phantom size: L, large, M, medium, S, small.

Figure 3 shows VBD values measured in the simulated phantoms. Figure 3a and b were generated without and with the skin correction, respectively, and these figures illustrate the importance of the aforementioned correction. Since the skin‐to‐breast tissue ratio depends on the total breast volume, if uncorrected, VBD measurements showed a volume‐dependent constant bias. Once the correction was implemented, as described in 2.1.2, the volume dependence was eliminated and the bias was reduced to −2%. The blue solid lines in Figure 3b indicate a ±10% difference in VBD, which is representative of the observed differences found in clinical studies comparing validated VBD measurement methods.^26^ Corrected and uncorrected measurements showed strong correlations (Pearson's r>0.95) to the nominal values.

In practical situations, the exact value of skin thickness is hard to determine. Therefore, an estimation of its impact on bias and variability was necessary. By varying the skin thickness within the 3−4 mm interval, we found that the bias ranged from −0.89% (for a skin thickness of 3 mm) to −3.2% (for a skin thickness of 4 mm). Similarly, the standard deviation of the differences between the nominal and measured values ranged from 1.28% (for 3 mm skin thickness) to 0.27% (for 4 mm skin thickness).

### 3.2 Breast density quantification in dual‐energy mammography clinical images and comparison to MRI measurements

**FIGURE 4:**
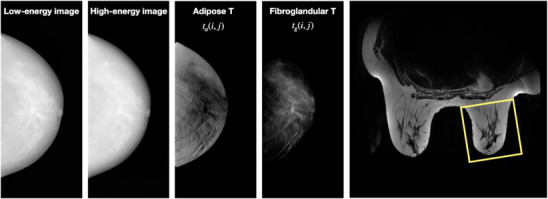
From left to right: LE and HE “for processing” mammograms, adipose tissue map, fibroglandular tissue map, MRI slice with a ROI delineating the analyzed breast (yellow rectangle). The measured VBD was 24.6% in mammograms and 29.0% in MRI. Hyper‐intense regions inside the MRI volumes of interest were considered adipose tissue.

Figure 4 illustrates the dual‐energy decomposition of a mammography clinical image pair, as well as an IDEAL MRI slice corresponding to the same patient.

**FIGURE 5:**
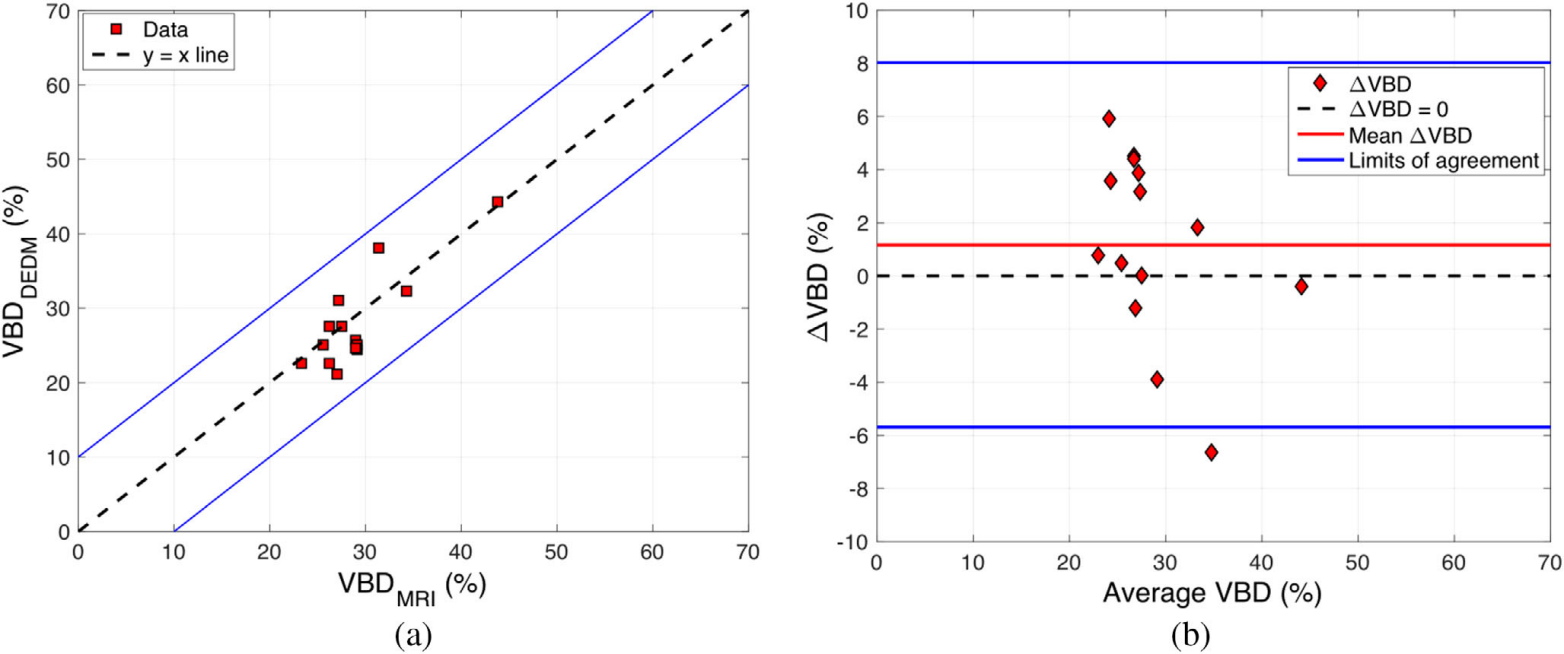
VBD values obtained in clinical dual‐energy and MRI images. (a) Scatterplot of $VBD_{DEDM}$ and $VBD_{MRI}$. The black dashed line shows the identity and blue solid lines indicate the ±10% difference margin. (b) Bland‐Altman plot comparing $VBD_{DEDM}$ and ${VBD}_{MRI}$. The black dashed line corresponds to no difference between the values, the red line indicates the mean in ΔVBD, and blue solid lines show the 95% limits of agreement.

Figure 5a shows the VBD obtained from the dual‐energy mammograms and MRI images. A moderate positive correlation between the densities measured in both modalities (ρ=0.64, p = 0.017) was found. Figure 5b is a Bland‐Altman plot comparing $VBD_{DEDM}$ and $VBD_{MRI}$. The differences were calculated as $\Delta VBD\;=\;VBD_{MRI}-VBD_{DEDM}$. The mean and median of the distribution of differences were 1.16% and 1.28%, respectively. The 95% limits of agreement were computed, as they provide an estimate of where the differences are expected to lie. The lower limit of agreement was −5.6%, while the upper limit of agreement was 8%. No trend was found in the differences, and the Wilcoxon signed rank test enabled to conclude that the median difference wasn't significantly different from zero.

## 4 DISCUSSION

The use of simulated phantoms with a completely known breast density has facilitated the assessment of the proposed method's intrinsic bias, as well as the validation of the proposed skin correction. The simulated images did not include x‐ray scattering and other properties of a real system, such as a space‐dependent detector response, and consequently, the presented simulation results represent a best‐case scenario for the method; results on a clinical system would approach that ideal once the aforementioned effects are accounted for.

Our simulation results show that, if a reasonable assumption of $t_{sk}$ is made, the proposed correction can account for the presence of the skin and produce accurate VBD measurements. Data from patient measurements show that the average total skin thickness (upper and lower breast surfaces) ranges from 3 to 4 mm,^19^, ^20^ so values within that interval should provide a good approximation. The effect of varying $t_{sk}$ was studied, and it was found that the correction is relatively stable in that interval. In a clinical application, a closer approximation of each patient's skin thickness could be derived from the projected image.

Currently, there is a lack of results assessing the performance of material decomposition in 2D mammograms using anthropomorphic phantoms (virtual or otherwise) as a reference. However, given the recent interest in dual‐energy digital breast tomosynthesis (DE‐DBT), some studies have assessed the material decomposition technique in DBT projections. Lu et al.^13^ used the material decomposition formalism to estimate VBD in the central projections of DE‐DBT acquisitions. They reported root mean square errors of 2.64% to 3.34% for uniform, variable thickness phantoms, 4.17% for uniform, variable density phantoms, and 4.49% for nonuniform, variable thickness phantoms. These values are consistent with our phantom studies and suggest the extendibility of our method to DBT projections. However, it is important to note that the phantoms used by Lu et al. did not feature a skin‐equivalent material and therefore do not fully capture the error's thickness dependence in real breasts.

Breast density quantified in central DBT projections has been found to be comparable to regular mammography breast density measurements,^27^ indicating that our method could be applied in DBT examinations as well. In addition to using only the central projection, a semi‐3D tissue separation in DE‐DBT can be achieved by material decomposition in the projection domain followed by reconstruction.

Recently, Kim et al.^28^ explored the application of an indirectly calibrated material decomposition method to DE‐DBT images with the aim of generating compositional contrast for cancerous masses. They proposed a low‐dose DE‐DBT acquisition technique and evaluated their method using anthropomorphic digital phantoms. However, their study did not specifically quantify VBD or report the potential impact of skin on the tissue‐specific images, as their focus was on contrast generation and the breast was assumed to consist of adipose, glandular, and cancerous mass tissues. It would be interesting to further investigate the performance of VBD quantification when material‐specific projections are used as inputs or parameters in different DE‐DBT reconstruction algorithms.

The acquisition technique used in our proof of concept was optimized for contrast medium visualization with a reasonable average glandular dose. Our results suggest that the implementation of the dual‐energy VBD method is viable for the images acquired in CEM patients. A custom‐made dual‐energy acquisition technique for compositional imaging could be optimized, but this would affect the clinical application since a separate dual‐energy study may not be justified for the sole purpose of VBD measurement.

To ensure robust results, we have chosen to exclude patients with breast implants or significant lesions from our study cohort. Breast implants can introduce distortions and alter the tissue's dual‐energy decomposition. Similarly, the presence of large lesions or tumors may introduce artifacts in VBD measurements. However, a study by Youk et al.^29^ found no statistically significant differences in VBD between patients with lesions and those without. This suggests that for small lesions, our method may still offer reliable VBD measurements.

Given that the clinical dual‐energy mammography images used in this study were acquired post‐contrast in CEM, the potential presence of iodine in the breast tissue could introduce bias in the density measurements. Our radiologists, familiar with CEM images, did not detect iodine uptake in any of the images, indicating that a potential iodine presence was below the detectable threshold of 0.5 mg/cm^2^. To address the potential impact of iodine on dense tissue quantification, we considered iodine as an additional material and performed its dual‐energy decomposition. If 0.5 mg/cm^2^ of iodine were present, it would result in a 4% increase in local fibroglandular tissue density for a 5 cm breast. The impact of this bias is modulated by the fraction of the area of the breast occupied with the potential iodine uptake. A background parenchymal uptake would keep the impact on the VBD close to 4%, while a small local uptake (around 10% of the whole breast) would lower the impact to 0.4%. Although it is not possible to know the extent of the iodine uptake below the detectability limit, large background parenchymal enhancement is unlikely. Gennaro et al.^30^ have quantified VBD in CEM images using Volpara, finding that 2% of the 150 analyzed CEM images showed marked background parenchymal enhancement. Based on these considerations, we believe that the potential minimal iodine present in the contralateral breast did not significantly affect our VBD quantification in this study.

As reported in previous multimodality studies, the overestimation of VBD relative to MRI and CT is a known feature of some mammography‐based methods.^31^, ^32^, ^33^, ^34^ As shown by our simulation, in dual‐energy techniques an overestimation in VBD could be partially attributed to the contribution of the skin to the tissue maps. Once this effect had been corrected, our $VBD_{DEDM}$ measurements showed no significant over‐estimation when compared to $VBD_{MRI}$. The disagreement between the two modalities fell under the ±10% margin.

The inclusion of MRI in our comparative analysis served as a reference point to assess the validity of our VBD quantification method in dual‐energy mammograms. While MRI is not a clinical “gold standard” to quantify VBD, it is a 3D modality with inherently strong soft tissue contrast, so even a simple quantification method can provide an accurate estimate of the volumetric distribution of fibroglandular tissue.

Rahbar et al.^34^ performed a three‐way comparison between two validated mammography‐based methods (Quantra and Volpara, both approved by the United States Food and Drug Administration) and MRI. Comparing the 2D methods to MRI, they found upper and lower limits of agreement of 7.4% and −8.2% (Volpara) and 14.9% and −7.0% (Quantra). The relative performance of 3D‐based modalities has also been investigated, and Chen et al.^35^ found upper and lower limits of agreement of 8.4% and −10.2% when comparing CT VBD measurements to MRI. The upper and lower limits of agreement in our comparison were 8% and −5.6%, showing that the proposed VBD method performs similarly to other validated mammography‐based methods when using MRI measurements as a reference.

Concerning other potential uses of the investigated technique, Pacheco et al.^23^ have utilized the total breast thickness maps obtained from the dual‐energy decomposition to estimate the background signal in CEM images. The precise estimation of the background signal is crucial to the isolation of iodine signal and consequently the generation of iodine concentration maps. Furthermore, certain implementations of the dual‐energy subtraction technique rely on a thickness‐dependent weight factor,^36^ and thus could benefit from the thickness maps generated by the dual‐energy decomposition. This shows a potential utility of dual‐energy decomposition beyond breast density assessment.

The ability to accurately estimate breast density in projections and the potential for application in low‐dose DBT screening scenarios highlights the clinical relevance of the dual‐energy decomposition technique, and consequently the current importance of further exploring these techniques.^13^, ^27^, ^37^ Our virtual phantom study has provided an upper bound for the quantitative performance of material decomposition in central projections.

## 5 CONCLUSIONS

We have assessed the intrinsic performance of a method for measuring VBD in dual‐energy digital mammograms. Our method could quantify VBD in simulated images of anthropomorphic phantoms with errors below 5%. We have presented an assessment of the method in a simulated environment, as well as a comparison to VBD quantification in MRI clinical images as a proof‐of‐concept. There was no statistical difference between dual‐energy mammography and MRI VBD measurements in the same patient group. In order to draw definite conclusions on the agreement between the two modalities, a larger sample size would be needed. Our evaluation using virtual phantoms provides an upper bound on the method's ability to accurately estimate VBD in realistic tissue distributions and highlights the importance of considering the skin when performing dual‐energy decomposition. The presented method and its assessment can be extended to tomosynthesis projections in order to assess the feasibility of projection‐domain material decomposition in DE‐DBT.

## AUTHOR CONTRIBUTIONS

All authors made substantial contributions to the conception and design of the work; the acquisition, analysis and interpretation of the data for the work; drafting and critical revision of the work for important intellectual content; and gave final approval of the submitted version.

## CONFLICT OF INTEREST STATEMENT

The authors have no relevant conflicts of interest to disclose.
